# FUNCTIONAL IMPACT ON ADULTS AND OLDER PEOPLE AFTER HOSPITALIZATION BY COVID-19

**DOI:** 10.1093/geroni/igad104.3364

**Published:** 2023-12-21

**Authors:** Gabriela Ochiai, Caroline De Godoy, Erika Gouveia E Silva, Celso Ricardo Fernandes De Carvalho, Carolina Fu, Clarice Tanaka, Carlos Roberto Ribeiro De Carvalho, Jose Pompeu

**Affiliations:** University of Sao Paulo, Sao Paulo, Sao Paulo, Brazil; University of Sao Paulo, Sao Paulo, Sao Paulo, Brazil; University of Sao Paulo, Sao Paulo, Sao Paulo, Brazil; University of Sao Paulo, Sao Paulo, Sao Paulo, Brazil; University of Sao Paulo, Sao Paulo, Sao Paulo, Brazil; University of Sao Paulo, Sao Paulo, Sao Paulo, Brazil; University of Sao Paulo, Sao Paulo, Sao Paulo, Brazil; University of Sao Paulo, Sao Paulo, Sao Paulo, Brazil

## Abstract

Cross-sectional study of 159 individuals, one month after hospitalization at the Hospital das Clínicas of the University of São Paulo for Covid-19, divided into groups: 54.7% (n=87) were adults (< 60 years), and 45.3% (n=72) were older people (≥60 years), with 43.4% (n=69) being female. Those who did not accept to participate, without availability, or without ability to understand the questionnaires were excluded. Functional capacity was assessed by the Barthel Index (BI). Statistical analysis was performed using JASP Statistics-0.15.0, considering significance level of alpha=0.05. Wilcoxon and Mann-Whitney tests were applied to compare pre- and post-Covid periods and to compare adults and older people, respectively. Data collection went from June 30, 2020, to August 24, 2021. The median length of hospitalization was 23 (15-34) days. The median total BI scores were lower one month after discharge in older people, 95 [90-100], when compared to adults, 100 [92.5-100] (p=0.001). Bladder control (p=0.009), toilet use (p=0.021), chair/bed transfers (p=0.043), ambulation (p=0.006), dressing (p=0.015), stair climbing (p< 0.001), and bathing self (p=0.006) were the activities of daily living most affected in older people. Hospitalization due to Covid-19 had functional impacts in the post-discharge period, particularly in older people, manifested by limitations in performance of daily activities with higher physical demand. These findings emphasize the need for functional capacity screening after hospitalization for Covid-19, especially in older people.

